# Technology-Critical Element Exposure Reveals Divergent Toxicity in Different Human Cells Despite Comparable Uptake

**DOI:** 10.3390/biom16010113

**Published:** 2026-01-08

**Authors:** Tudor-Mihai Magdaș, Gabriela Adriana Filip, Adriana Dehelean, Simona Clichici, Constantin Bodolea, Andrei Mihai Bălan, Dana Alina Magdaș, Carmen Bianca Crivii, Ioana Bâldea

**Affiliations:** 1 Anesthesia and Intensive Care Department, “Iuliu Hatieganu” University of Medicine and Pharmacy, 8 Victor Babeş Street, 400012 Cluj-Napoca, Romania; tmmagdas@gmail.com (T.-M.M.); constantin.bodolea@umfcluj.ro (C.B.); balan.andrei@umfcluj.ro (A.M.B.); 2 National Institute for Research and Development of Isotopic and Molecular Technologies, 67-103 Donat Street, 400293 Cluj-Napoca, Romania; adriana.dehelean@itim-cj.ro (A.D.); alina.magdas@itim-cj.ro (D.A.M.); 3 Department of Anatomy and Embryology, “Iuliu Hațieganu” University of Medicine and Pharmacy, 8 Victor Babeș Street, 400012 Cluj-Napoca, Romania; bianca.crivii@umfcluj.ro; 4 Department of Physiology, Faculty of Medicine, “Iuliu Hatieganu” University of Medicine and Pharmacy, 400347 Cluj-Napoca, Romania; sclichici@umfcluj.ro (S.C.); ioana.baldea@umfcluj.ro (I.B.); 5 Department of Anaesthesia and Intensive Care, Municipal Clinical Hospital, 400139 Cluj-Napoca, Romania

**Keywords:** technology-critical elements, rare earth elements, cytotoxicity, oxidative stress, cellular uptake

## Abstract

The increasing use of Technology-Critical Elements (TCEs) in modern technology has led to widespread environmental release, raising questions about their biological effects, as emerging evidence suggests significant toxicity. We investigated the effects of three technology-critical elements, Indium oxide (In_2_O_3_), Lanthanum nitrate hexahydrate (La(NO_3_)_3_·6H_2_O) and Cerium(III) nitrate hexahydrate (Ce(NO_3_)_3_·6H_2_O), on human dermal fibroblasts (BJ) and hepatocarcinoma cells (HepG2), assessing their uptake, impact on viability, and induced cellular stress responses, quantified by markers of inflammation, oxidative stress, and membrane damage. Our results show a strong differential susceptibility: normal BJ fibroblasts proved vulnerable, whereas HepG2 cells were highly resistant. This divergence occurred despite substantial and comparable accumulation of all three TCEs in both cell lines, indicating that toxicity is uncoupled from the magnitude of the uptake. Mechanistically, the differential toxicity correlated strongly with opposing antioxidant responses. Additionally, low concentrations of cerium (III) nitrate (12.5–50 µg/mL) uniquely stimulated the proliferation of HepG2 cells (up to 129% of control). While these findings identify multiple mechanistic hazards regarding the potential of low-level technology-critical element exposure, they must be interpreted cautiously and warrant further investigation in more complex biological models.

## 1. Introduction

Modern society is increasingly reliant on a group of elements known as Technology-Critical Elements (TCEs), which are indispensable for manufacturing components for a wide array of advanced technologies [1,2,3]. However, the global supply chain for these materials is fraught with risk, as it is heavily concentrated in a few nations and subject to geopolitical tensions and market volatility [3]. Significant environmental concerns arise, as the complete lifecycle of these elements—from mining and industrial processing to disposal of e-waste results in their emergence as widespread environmental contaminants [1,2,4,5], with projections of future increases in global waste generation, of which only a fraction is expected to be properly collected and recycled [6]. Furthermore, the industrial “balance problem”, where low-value but high-volume elements like Lanthanum (La) and Cerium (Ce) are produced as byproducts, creates an incentive for new applications that increase their environmental release [7]. TCEs have been confirmed in environmental compartments from which they can enter the food chain, a main route of exposure for the general population, in addition to other pathways, including inhalation of aerosols, dermal contact, and iatrogenic routes, resulting in human exposure that is no longer limited to occupational settings [8,9,10,11,12].

While some TCEs are regulated under international frameworks, such as the EU Water Framework Directive (2000/60/CE and 2008/56/CE), the toxicological impacts of the “less-studied” TCEs, including Indium (In), remain poorly understood [13]. The available data indicate conflicting and often opposing biological outcomes, driven by the specific element, its chemical speciation, and the cellular context [14,15,16].

Available data have revealed that TCEs induce multisystemic damage in humans and animal models, affecting the respiratory, cardiovascular, nervous, and reproductive systems. Multiple mechanisms for toxicity have been implicated, ranging from the direct antagonism of critical calcium channels, due to similarities in ionic radii of lanthanides (Ln^3+^) with calcium (Ca^2+^), to the generation of reactive oxygen species (ROS) and oxidative stress, inflammation pathway activation, and mitochondrial dysfunction [8,17,18,19].

Further complicating the already complex picture, Rare Earth Elements (REEs) are known to induce a hormetic effect, in which exposure to low doses promotes growth, while higher doses become inhibitory [20]. This stimulatory effect has led to their use in agriculture and zootechnology, as these effects were observed across diverse biological kingdoms, from marine invertebrates to plants, fungi, or cyanobacteria [17,21,22,23,24,25]. In this context, the question of whether this observed hormetic phenomenon extends to human cells, potentially stimulating mitogenic activity or causing disease, warrants further investigation [23].

We selected an experimental concentration range of 12.5–400 µg/mL (29–913 µmol/L for the respective compounds) to establish a comprehensive dose–response relationship, a crucial step in the foundational toxicology of emerging contaminants [12,26,27]. The REEs exposure is an everyday occurrence primarily through the diet, which is the main pathway for the non-occupationally exposed individuals [9]. While current dietary intake (EDI) is typically low, with mean values reported at 1–2 µg/kg bw/day for the general Chinese population [28], and low internal concentrations are reported (e.g., median serum Cerium of 0.006 ng/mL in children) [27], the most exposure to REEs through diet is to Cerium and Lanthanum. The sum of REEs considered acceptable for a daily intake was proposed by Chinese researchers in the late 1990s, estimated at 70 µg/kg bw/day [29]. This reference value, which was derived from health surveys in Chinese REE mining areas and supporting animal data, does not represent a universally established international regulatory limit.

Currently, neither international nor European Union (EU) regulations have established a specific, universally applicable Acceptable Daily Intake (ADI) or Tolerable Daily Intake (TDI) for REEs in general human consumption. Multiple EU agencies manage the handling of these elements through regulations concerning Workplace and Environmental Safety [30], and controlling impurities via European guidelines for pharmaceuticals, though REEs are not explicitly listed [31]. This regulatory gap is critical because the use of REEs is expected to increase in the future [1,32], and given that REEs are emerging pollutants, increased environmental concentrations are probable [9]. Furthermore, increased REEs exposure is associated with multiple systemic diseases in humans [15]. On the other hand, long-term studies regarding the effects of chronic exposure are currently lacking, therefore preventing accurate estimation and exploration of cumulative doses. While environmental exposure occurs with multiple TCEs simultaneously, it is necessary to explore and understand the mechanisms of toxicity for each element individually and as a mixture.

The employed experimental concentrations (12.5–400 µg/mL) were titrated to find the target dose needed for IC50 determination and mechanistically investigate the toxic and hormetic (proliferative) effects of these elements, including the intracellular accumulation. We acknowledge that currently the general population is not exposed to acutely “toxic” or supraphysiological doses. Therefore we chose this concentration range aiming to gather foundational data in exploring these elements’ toxicity, as available data regarding their toxicology is scarce and often conflicting [9]. Determining the toxic doses and maximum EDI is critical for a prospective health risk assessment [9].

To address how the toxicological outcome is influenced by the distinct cellular phenotypes following TCE exposure, we evaluated the cytotoxicity, cellular uptake, and mechanisms of toxicity in normal dermal fibroblasts (BJ) and human hepatocarcinoma cells (HepG2) following exposure to Indium (as Indium(III) oxide, In_2_O_3_), Lanthanum (as Lanthanum(III) nitrate hexahydrate, La(NO_3_)_3_·6H_2_O), and Cerium (as Cerium(III) nitrate hexahydrate, Ce(NO_3_)_3_·6H_2_O).

## 2. Materials and Methods

### 2.1. Reagents and Cell Culture Medium

The cells used were normal dermal fibroblasts (BJ-CRL 2522- ATCC, Gaithersburg, MD, USA) and human hepatocarcinoma cells (HepG2-HB-8065, ATCC). They were cultivated in Dulbecco’s modified Eagle medium (DMEM) supplemented with 5% fetal calf serum (FCS), streptomycin, penicillin, and amphotericin in standard culture conditions. The medium was changed twice a week. The identical supplemented medium was used for both control and experimental groups. All reagents were purchased from Biochrom AG, Berlin, Germany. HepG2 cells are frequently used as an in vitro model for testing the impact on the liver cells [33].

The technology-critical elements were obtained from Merck (Sigma-Aldrich, Saint Louis, MO, USA) as follows: Indium(III) oxide (In_2_O_3_, 99.998% trace metals basis, yellow powder), Lanthanum(III) nitrate hexahydrate (La(NO_3_)_3_⋅6H_2_O, 99.999% trace metals basis, white powder or crystals), and Cerium(III) nitrate hexahydrate (Ce(NO_3_)_3_⋅6H_2_O, 99% trace metals basis, white to yellow powder or crystals). Indium oxide was handled as a micro-particulate suspension and the lanthanide nitrate salts were soluble in the culture medium.

### 2.2. Cytotoxicity Assessment

For the toxicity assay, cells (BJ and HepG2) were cultivated on 96-well plates at a density of 5 × 104 cells/well and accommodated for 24 h, then exposed to different concentrations of each sample suspended in fresh medium, ranging from 12.5 to 400 μg/mL (0.045–1.440 mmol/L for In_2_O_3_, from 0.029 to 0.924 mmol/L for La(NO_3_)_3_⋅6H_2_O, and from 0.029 to 0.921 mmol/L for Ce(NO_3_)_3_⋅6H_2_O). Solutions were made immediately before the exposure. Cells were then washed, and toxicity was assessed by using the neutral red toxicology assay kit (TOX4 1KT, Sigma Aldrich, Co., Heidelberg, Germany) as indicated by the producer. This assay allows for the estimation of the viable cells, which incorporate the dye, rather than the non-viable cells. The method is also included in the 3T3-NRU-phototoxicity-assay (OECD Guideline No. 432) guideline test [33].

Absorbance was read using an ELISA plate reader at 540 nm. All experiments were done in triplicate. Cells exposed only to medium were used as controls. Toxicity is expressed as a percentage of untreated controls. To complete the toxicity evaluation, the Lactate Dehydrogenase (LDH) was measured from the cell supernatant by using the LDH Cytotoxicity Colorimetric Assay Kit (E-BC-K771-M), purchased from ELab Science, Wuhan, China, as indicated by the producer.

### 2.3. Cellular Uptake Analysis

The cells were exposed to either medium (control group) or different metal concentrations in medium (fibroblasts: In-200 μg/mL, La-75 μg/ mL, and Ce-75 μg/ mL) and (HepG2: In-200 μg/ mL, La-200 μg/ mL, and Ce-75 μg/ mL)-treated groups. At the end of the 24 h of exposure, the cell supernatant was collected. The metal content was measured; results are presented as metal concentrations (μg/ mL) for each experimental group. Cell uptake was assessed as the difference between the amount of metal exposure and the remaining metal after treatment (measured in the supernatant).

Following metal exposure, cells were subjected to lysis on ice by using Igepal 0.1% and a protease inhibitor cocktail from plants 0.1% (Sigma Aldrich) in PBS for 1 h, then centrifuged. The supernatant was further used for biochemical analysis. The cell sediment was collected to measure the cell metal content, which was then compared to the indirect evaluation of the cell uptake based on the metal remaining in the cell medium. Data are expressed as μg/g of dried sediment.

An inductively coupled plasma mass spectrometer (ICP-MS, ELAN DRC (e), Perkin Elmer, USA) was used to detect the concentrations of La, Ce, and In in the cell supernatant and the cell sediment, respectively. The amount of REEs was quantified after complete mineralization of all samples with 2 mL of nitric acid (HNO3, 65% *v*/*v*) and 1 mL of hydrogen peroxide (H_2_O_2_, 30% *v*/*v*) in a closed-vessel microwave digestion system (Speed ENTRY by Berghof^®,^ Germany). After digestion, the resulting clear solution was diluted with ultrapure water to a final volume of 25 mL. For ICP-MS analysis, a Multi-element Calibration Standard 2 (PerkinElmer Pure Plus, USA, 10 μg/mL) and a Multi-element Calibration Standard 3 (PerkinElmer Pure Plus, USA, 10 μg/mL) were used to prepare standard stock solutions. All calibration curves showed strong linearity (regression coefficients, R > 0.999). The instrumental detection limits (LODs) were 0.2 ng/L (La) and 0.4 ng/L (Ce and In), and the quantification limits (LOQs) were 0.6 ng/L (La) and 1.2 ng/L (Ce and In), respectively.

### 2.4. Oxidative Stress and Inflammation Assessment

Following cell lysis, protein concentrations were measured using the DC assay kit according to the manufacturer’s specifications (Biorad, Hercules, CA, USA), using bovine albumin as a standard. For all assays, the lysates were corrected by total protein concentration. To assess the oxidative stress induced by exposure of the cells to metals, we measured the antioxidant defense enzyme activities, superoxide dismutase (SOD) and catalase (CAT), as well as malondialdehyde (MDA), a marker of lipid peroxidation due to oxidative stress-induced damage. The enzymatic activity of SOD was measured by using the Total Superoxide Dismutase (T-SOD) Activity Assay Kit (Hydroxylamine Method) (E-BC-K019-S), for Catalase the Activity Assay Kit (E-BC-K031-S) was used, MDA was measured using the malondialdehyde (MDA) Colorimetric Assay Kit (TBA Method) (all from Elab Science, Wuhan, China). Inflammatory markers, such as soluble interleukin 1β (IL-1β) and interleukin 6 (IL-6) were measured by using the ELISA Human Immunoassay kits (Elab Science, Wuhan, China). Cell supernatants for interleukins and cell lysates for SOD, CAT, and MDA were treated according to the manufacturer’s instructions; readings were performed at 450 nm with the correction wavelength set at 540 nm, using an ELISA plate reader (Tecan). The results are presented as Units/mg protein for SOD and CAT, nM/mg protein for MDA, pg/mL for IL-6 and OD/mL for IL-1β.

### 2.5. Statistical Analysis

Data are presented as mean ± standard deviation (SD). The cell viability data were analyzed using a two-way analysis of variance (ANOVA) to assess the main effects of cell type and metal compound concentration. The 20% inhibitory concentration (IC20) and half-maximal inhibitory concentration (IC50) were used to quantify and compare the cytotoxic effects across the two cell lines to enable a quantitative comparison for compounds. The IC20 and IC50 were determined for each compound and cell line by fitting the dose–response data to a four-parameter logistic (4PL) non-linear regression model using GraphPad PRISM statistical software, version 5. For the toxicology (LDH), oxidative stress (SOD, CAT, MDA), and inflammation assays (IL-6, IL-1β), the effects of metal exposure were analyzed separately within each cell. Statistical significance for all tests was set at *p* < 0.05.

## 3. Results

The aim of this work was to firstly test the toxicity potential of three TCEs, namely Ce, La, and In, on two types of cells represented by normal dermal fibroblasts (BJ) and one tumoral, namely human hepatocarcinoma cells (HepG2). The compounds that were involved in this test were indium oxide (In2O3) along with cerium and lanthanum nitrates.

### 3.1. Cell Viability

First, cell viabilities of the normal dermal fibroblasts (BJ) and human hepatocarcinoma cells (HepG2) were assessed in response to varying concentrations of indium oxide, cerium (III) nitrate hexahydrate, and lanthanum (III) nitrate hexahydrate.

**Figure 1:**
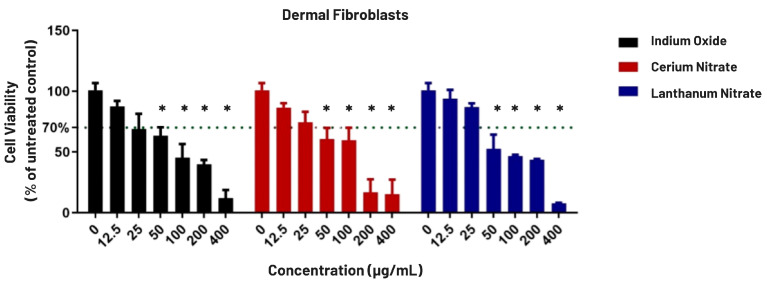
Viability of normal dermal fibroblasts (BJ) following 24 h exposure to technology-critical elements. Error bars represent the mean ± SD (n = 3). The green dotted line indicates the 70% viability threshold. Statistical significance compared to the un-treated control was determined by two-way ANOVA: (*) represents a significant reduction in viability (*p* < 0.05).

**Figure 2:**
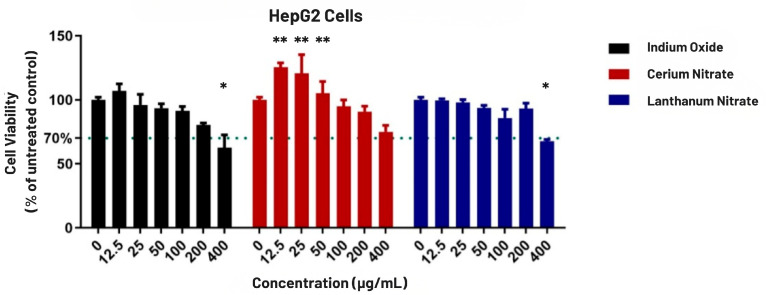
Viability of human hepatocarcinoma cells (HepG2) following 24 h exposure to technology-critical elements. Error bars represent the mean ± SD (n = 3). The dotted line indicates the 70% viability threshold. Statistical significance compared to the untreated control was determined by two-way ANOVA: (*) represents a significant reduction in viability (*p* < 0.05), while (**) indicates a significant proliferative (hormetic) effect (*p* < 0.01).

A pronounced toxic effect was observed in the dermal fibroblast cells, with a dose-dependent reduction in cell viability (Figure 1). The toxic effects were observed particularly at concentrations higher than 50 µg/mL. In contrast, the malignant HepG2 cells were markedly resistant (Figure 2). At lower concentrations (12.5 to 50 µg/mL), Cerium induced a significant increase in cell viability, reaching up to 129% of the untreated control, while at higher concentrations (>100 µg/mL), this proliferative effect subsided, and a modest toxic response was observed.

The results obtained from applying two-way ANOVA to each metal dose showed significant differences in cell viability between the two types of tested cell lines, regardless of the administered compound (indium (III) oxide, cerium (III) nitrate hexahydrate, and lanthanum (III) nitrate hexahydrate) or its concentration. The results of the two-way ANOVA, which confirm a significant difference in cell viability between the two cell lines across all tested compounds and concentrations, are available in Supplementary Table S1.

To quantify the differential cytotoxicity observed in the viability assays, half-maximal inhibitory concentration (IC_50_) and 20% inhibitory concentration (IC_20_) for each compound were determined. Normal dermal fibroblasts were markedly more sensitive to all three metals, exhibiting a clear, dose-dependent reduction in cell viability (Figure 1 and Figure 2). Lanthanum nitrate exhibited the highest toxicity, with an IC_50_ of 52.88 µg/mL, followed by Indium oxide at 69.04 µg/mL and Cerium nitrate at 69.53 µg/mL (Table 1).

Conversely, the malignant HepG2 cell line demonstrated marked resistance to all tested compounds. For all three metals, cell viability remained above 50%, even at the highest concentration of 400 µg/mL, so IC_50_ values were not reached. Notably, due to the proliferative (hormetic) effect of Cerium at low concentrations in HepG2 cells, a 20% reduction in viability was never achieved, making the IC_20_ value for this specific condition incalculable.

The impact on cellular integrity, measured by LDH release into the medium, further illustrated the differential responses. In fibroblasts, a significant effect of treatment was observed. Lanthanum (*p* < 0.05) and Cerium (*p* < 0.05) significantly increased LDH release, indicating substantial membrane damage. Notably, Indium exposure resulted in a significant decrease in LDH release compared to the fibroblast control (*p* < 0.0001). In HepG2 cells, the response was uniformly protective; all three metals significantly decreased LDH release compared to the baseline HepG2 control (*p* < 0.0001).

### 3.2. Cellular Uptake

Cellular uptake of In, La, and Ce was measured following a 24 h exposure at sublethal concentrations. Both fibroblasts and HepG2 cells presented significant accumulation of all three metals (Table 2 and Table 3), indicated by metal depletion in the culture medium and the high amounts of metals detected in the insoluble cell sediment, representing the fraction bound to the cellular structures after washing and cell lysis (Table 2 and Table 3).

Based on the culture medium analysis, Indium uptake was strongly enhanced in both cell lines (>98%). Cerium uptake was lower (>81%). Lanthanum uptake was the lowest among the 3 metals, with 62.39% for fibroblasts and 74.79% for HepG2 cell. The amount of La administered was different between the fibroblasts (75 μg/mL) and hepatic cells (200 μg/mL), in accordance with the calculated IC50 for each cell line.

Analysis of the insoluble cell sediment also confirmed the substantial intracellular localization estimated before. Dry weight for Indium reached levels of nearly 7 × 104 µg/g in both dermal fibroblasts ((6.97 ± 0.01) × 10^4^ µg/g) and HepG2 cells ((6.69 ± 0.01) × 10^4^ µg/g). Lanthanum reached levels of (3.64 ± 0.08) × 10^3^ µg/g in fibroblasts and with (5.83 ± 0.05) × 10^3^ in HepG2 cells, while Cerium reached (3.68 ± 0.10) × 10^3^ µg/g in dermal fibroblasts and (2.70 ± 0.05) × 10^3^ µg/g in HepG2 cells.

The substantial cell-associated metal load was comparable across both cell lines, demonstrating a direct correlation between the two distinct measurement methods: the clearance of metals from the extracellular environment and the quantification of metals bound to cellular structures within the dry sediment fraction.

### 3.3. Oxidative Stress and Proinflammatory Markers Evaluation

Following exposure to the compounds, namely indium oxide 200 µg/mL, cerium (III) nitrate hexahydrate 75 µg/mL, and lanthanum (III) nitrate hexahydrate 200 µg/mL, for HepG2 and 75 µg/mL for BJ fibroblasts, we assessed key markers of oxidative stress (SOD, CAT, MDA) and inflammation (IL-6, IL-1β). The results revealed fundamentally different and opposing cellular stress responses to these three metals (Table 4 and Table 5). A comparative summary of these effects has been included in the Supplementary Materials (Supplementary Table S2).

All three tested metals suppress SOD activity in fibroblasts (One-way ANOVA, *p* < 0.05) compared to controls, while HepG2 cells mounted an adaptive response, inducing the significant upregulation of SOD activity in the hepatocarcinoma cells (In, *p* < 0.001; La, *p* < 0.01; Ce, *p* < 0.001).

Catalase (CAT) activity showed cell-specific changes. In fibroblasts, exposure to Lanthanum caused significantly decreased activity (*p* < 0.01), while Cerium significantly increased it (*p* < 0.001). No significant changes in CAT activity were observed in HepG2 cells after exposure to these three elements.

Analysis of malondialdehyde (MDA) revealed complex metal-specific effects. In fibroblasts Indium significantly increased MDA levels (*p* < 0.001), whereas Lanthanum (*p* < 0.05) and Cerium (*p* < 0.001) decreased it. In the HepG2 cells, the pattern was distinct, with Lanthanum significantly increased MDA (*p* < 0.001), while Indium (*p* < 0.01) and Cerium (*p* < 0.001) decreased MDA levels.

The inflammatory response was assessed via analysis of IL-6 and IL-1β levels. In fibroblasts Indium significantly elevated IL-6 levels (*p* < 0.05), while Cerium suppressed it (*p* < 0.01). In HepG2 cells, Indium and Cerium both significantly decreased IL-6 (*p* < 0.0001), whereas Lanthanum markedly increased it (*p* < 0.0001). Effects on IL-1β were modest, with no significant effects observed in fibroblasts. In HepG2 cells, the effect was statistically significant. Thus, Indium increased (*p* = 0.0055) and Cerium decreased (*p* = 0.0410) the cytokine’s secretion in the supernatant. Collectively, these data generate the hypothesis that the cytotoxic and inflammatory potential of these metals is highly cell-type dependent.

## 4. Discussion

**Figure 3:**
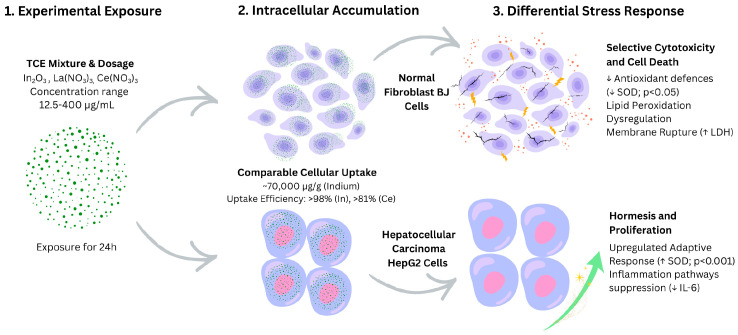
Divergent toxicological responses of normal vs. malignant cells following technology-critical element (TCE) exposure.

The current study investigated the cellular responses of two distinct cell lines, normal fibroblasts (BJ) and hepatocarcinoma cells (HepG2), to three key TCEs (In, La and Ce). Our results demonstrated that the cellular phenotype is the primary determinant of the biological behaviour. Despite substantial uptake in both cell types, viability outcomes were divergent and correlated with opposing antioxidant enzyme responses (Figure 3).

Analysis of the cell sediment confirmed substantial metal accumulation in both cell lines (Table 3). For instance, following exposure to Indium oxide, both cell lines demonstrated high indium uptake (>98%), but the viability outcomes were opposite. We must acknowledge that this measurement represents the total cell-associated fraction. Due to the potential for extracellular binding or surface precipitation, a portion of this load may be sequestered on the cell membrane or adsorbed to the culture vessel than being fully internalized. Nevertheless, even if a fraction of the metal remained extracellular, the total cell-associated accumulation was comparable between the cell lines for every metal. Thus, the divergent viability outcomes between fibroblasts and HepG2 cells indicate that the cellular phenotype is the decisive factor in the toxicological response.

The high concentration of metals retrieved from the insoluble cell sediment following cell lysis indicates significant intracellular immobilization, possibly due to binding to intracellular components or within organelles. Also, the unexpected cellular behavior indicates that the toxicological impact is determined after internalization, possible by the interactions of the metals with diverse intracellular structures. This divergence indicates that the handling of these elements is fundamentally different between cell lines, with the toxicological impact likely determined after internalization through interactions with di-verse intracellular structures.

The ability of the cells to manage the internalized TCE load appears to be linked to their antioxidant systems, as the different toxicity between BJ and HepG2 cells is correlated with different behavior of the pro-oxidant/antioxidant balance. In normal fibroblasts, exposure to all three TCEs was associated with a significant decrease in SOD activity, while HepG2 cells response was associated with a significant increase in the SOD activity and heightened viability. However, it is important to acknowledge that the changes regarding antioxidant activity may represent a stress response, rather than being the primary driver of the viability outcome.

In normal fibroblasts, indium induced a pro-oxidant and pro-inflammatory effect, while in HepG2 cells, it induced mixed responses, suppressing IL-6 and increasing IL-1β. We observed an apparent discrepancy between viability and membrane damage. In fibroblasts, Indium caused a significant viability loss, but it induced minimal membrane disruption, as reflected by minimal LDH release. Conversely, Lanthanum and Cerium exposure led to important LDH release, suggesting significant membrane disruption. In HepG2 cells, all three metals significantly decreased LDH release compared to the control, possibly reflecting an adaptive response. These findings suggest that indium toxicity may be associated with non-lytic pathways of cell death, such as apoptosis, whereas lanthanides induce an effect dominated by direct membrane disruption [34,35,36]. These findings, which suggest that the co-occurring TCEs exert cytotoxic effects through multiple, distinct pathways, raise concerns about whether a mixture of these elements could exhibit heightened toxicity through synergistic actions –a scenario that more accurately reflects real-world environmental exposures.

Oxidative stress is only one part of the multi-faceted TCE-associated toxicity, as multiple other pathways, including apoptosis, autophagy, or the ability of lanthanides to competitively bind calcium-dependent molecules due to similarities in ionic radii, likely contribute to the broad toxicological picture [18,19,37,38,39]. Our results contrast with the available data in the literature, as previous studies reported complex REE-based organic salts or indium inorganic compounds as being largely non-toxic to fibroblasts or HepG2 cells [34,35]. These discrepancies highlight the role of both chemical speciation and the specific cellular phenotype. Furthermore, the specific culture medium itself is considered an important factor, as its composition affects the aggregation, dissolution, and bioavailability of metal compounds [40]. Indium oxide (In_2_O_3_) has distinct physicochemical variables comparing to lanthanide salts—nitrate salts dissociate into free ions, while Indium oxide remains largely insoluble. The insoluble metal particulates enter the cells via endocytic pathways rather than ionic channels [41]. A difference in the internalization level was also reflected in our analysis, with particulate Indium exhibiting higher cellular uptake (>98%) comparing to the soluble salts.

Cerium nitrate presented a hormetic effect in HepG2 cells by promoting cellular proliferation at low concentrations (12.5–50 µg/mL), while higher concentrations induced a cytotoxic response [42]. The proliferative effect in the cancer cell line raises concerns regarding a potential role of low-level exposure in carcinogenesis. The experimental concentrations are supraphysiological compared to current human exposure levels and therefore these results do not suggest immediate evidence of risk and any translation to living organisms remains speculative [27,28,29]. We acknowledge that in vitro toxicity data is foundational and is not directly translatable to in vivo scenarios, but such data remains essential for addressing the knowledge gaps required for the future development of monitoring strategies and regulations of these emerging contaminants [5].

## 5. Conclusions

This study demonstrates that the toxicity of Indium, Lanthanum, and Cerium is primarily determined by the cellular phenotype. Dermal fibroblasts and hepatocellular carcinoma cells accumulated substantial and comparable amounts of each metal, but the biological effects diverged significantly. This difference in the biological behavior also correlated with opposing modulatory effects on the antioxidant defense systems.

These in vitro results represent foundational toxicological data and provide mechanistic insights necessary for understanding the complex picture of TCE-associated toxicity. As global reliance on advanced technologies is increasing, it is paramount to address the biological effects of these emerging contaminants, to ultimately develop environmental safety frameworks.

## Figures and Tables

**Table 1:** Cytotoxicity of TCE compounds expressed as 20% (IC_20_) and 50% (IC_50_) inhibitory concentrations (µg/mL). * Incalculable due to hormetic proliferation at low concentrations.

Compound	BJ Dermal Fibroblast		HepG2	
	IC20	IC50	IC20	IC50
Indium Oxide	23.26	69.04	247.93	>400
Cerium Nitrate	27.60	69.53	Incalculable *	>400
Lanthanum Nitrate	35.25	52.88	291.68	>400

**Table 2:** Concentration of the metals measured in the cell supernatant (medium). Data are presented as mean ± SD.

Fibroblast Supernatant				HepG2 Supernatant		
Metal	Control (μg/mL)	Treated (μg/mL)	Cell uptake (μg/mL)	Control (μg/mL)	Treated (μg/mL)	Cell uptake (μg/mL)
Indium	0.04± 2.5 × 10^−4^	3.15± 3.33 × 10^−2^	196.84	0.098± 4 × 10^−3^	1.63± 8.67 × 10^−2^	198.37
Lanthanum	0.001 ± 7.21 × 10^−5^	17.31 ± 0.42	57.68	0.018± 3.51 × 10^−3^	50.52± 0.871	149.58
Cerium	0.002± 3.04 × 10^−4^	12.15± 0.237	62.85	0.092± 6 × 10^−3^	14.41± 0.79	60.58

**Table 3:** Concentration of the metals measured in the cell sediment (after membrane lysis). Data are presented as mean ± SD.

Fibroblast Sediment			HepG2 Sediment	
Metal	Control (μg/g)	Treated (μg/g)	Control (μg/g)	Treated (μg/g)
Indium	2.79 ± 0.21	(6.97 ± 0.01) × 10^4^	59.47 ± 1.46	(6.69 ± 0.01) × 10^4^
Lanthanum	(4.5 ± 20) × 10^−3^	(3.64 ± 0.08) × 10^3^	2.25 ± 0.04	(5.83 ± 0.05) × 10^3^
Cerium	(7.9 ± 40) × 10^−3^	(3.68 ± 0.10) × 10^3^	7.71 ± 0.35	(2.70 ± 0.05) × 10^3^

**Table 4:** Effects of Indium (In), Lanthanum (La), and Cerium (Ce) on redox imbalance and inflammation in fibroblasts. Data are presented as mean ± SD.

Treatment	Interleukin-6 (IL-6) (pg/mL)	Interleukin-1β (IL-1β) (OD/mL)	Malondialdehyde (MDA) (nM/mg Protein)	Superoxide Dismutase (SOD) (U/mg Protein)	Catalase (CAT) (U/mg Protein)
Control	285.04 ± 10.61	0.035 ± 0.001	0.429 ± 0.026	35.96 ± 1.62	19.96 ± 1.01
Indium (In)	329.66 ± 14.88	0.034 ± 0.002	0.566 ± 0.025	26.51 ± 2.28	21.59 ± 1.76
Lanthanum (La)	269.85 ± 15.67	0.040 ± 0.002	0.394 ± 0.035	30.52 ± 1.91	16.95 ± 1.13
Cerium (Ce)	224.35 ± 10.31	0.035 ± 0.002	0.336 ± 0.025	31.55 ± 2.26	24.58 ± 1.41

**Table 5:** Effects of Indium (In), Lanthanum (La), and Cerium (Ce) on redox imbalance and inflammatory markers in HepG2 cells. Data are presented as mean ± SD.

**Treatment**	**Interleukin-6 (IL-6)** **(pg/mL)**	**Interleukin-1β (IL-1β)** **(OD/mL)**	**Malondialdehyde (MDA)** **(nM/mg Protein)**	**Superoxide Dismutase (SOD)** **(U/mg Protein)**	**Catalase (CAT)** **(U/mg Protein)**
Control	144 ± 10.74	0.031 ± 0.001	0.531 ± 0.056	29.46 ± 3.19	24.18 ± 1.90
Indium (In)	89.93 ± 4.83	0.037 ± 0.002	0.433 ± 0.055	46.50 ± 2.44	20.65 ± 1.85
Lanthanum (La)	215.40 ± 10.17	0.032 ± 0.001	0.681 ± 0.036	40.48 ± 1.71	19.35 ± 1.40
Cerium (Ce)	95.33 ± 9.76	0.029 ± 0.002	0.387 ± 0.041	42.06 ± 3.39	22.67 ± 2.29

## Data Availability

The original contributions presented in this study are included in the article/Supplementary Material. Further inquiries can be directed to the corresponding author.

## Supplementary Materials

The following supporting information can be downloaded at https://www.mdpi.com/article/10.3390/biom16010113/s1. Supplementary Table S1. Two-way ANOVA results corresponding to the viability of the tested cell lines (Fb and HepG2) at a given concentration of administered compound (indium (III) oxide, cerium (III) nitrate hexahydrate, and lanthanum (III) nitrate hexahydrate). Supplementary Table S2. A comparative summary of the effects of Indium (In), Lanthanum (La), and Cerium (Ce) on key markers.

## Abbreviations

The following abbreviations are used in this manuscript:

ADI	Acceptable Daily Intake
CAT	Catalase
Ce	Cerium
DMEM	Dulbecco’s Modified Eagle Medium
EDI	Estimated Dietary Intake
ELISA	Enzyme-Linked Immunosorbent Assay
EU	European Union
IC_20_	20% Inhibitory Concentration
IC_50_	50% Inhibitory Concentration
ICP-MS	Inductively Coupled Plasma Mass Spectrometry
IL-1β	Interleukin 1β
IL-6	Interleukin 6
In	Indium
La	Lanthanum
LDH	Lactate Dehydrogenase
MDA	Malondialdehyde
REEs	Rare Earth Elements
ROS	Reactive Oxygen Species
SD	Standard Deviation
SOD	Superoxide Dismutase
TCEs	Technology-Critical Elements
TDI	Tolerable Daily Intake
