# Supplementary material for: Technology-Critical Element Exposure Reveals Divergent Toxicity in Different Human Cells Despite Comparable Uptake
Source: Biomolecules. 2026 Jan 8;16(1):113. doi: 10.3390/biom16010113 (PMC12839252; doi:10.3390/biom16010113)
Supplement: Supplementary file 1 [file biomolecules-16-00113-s001.zip › biomolecules-4065129-supplementary.pdf]

**Technology-Critical Element Exposure Reveals Divergent Toxicity in Different Human Cells Despite Comparable Uptake**

**Supplementary Table S1.** Two-way ANOVA results corresponding to the viability of the tested cell lines (Fb and HepG2) at a given concentration of administered compound (indium (III) oxide, cerium (III) nitrate hexahydrate, and lanthanum (III) nitrate hexahydrate).

| **Concentration (µg/ml)** | **P value** | | |
|---|---|---|---|
| | Row factor (cell type) | Column factor (compound) | Interaction |
| **12.5** | **<0.0001** | **0.0074** | **0.0002** |
| **25** | **<0.0001** | **0.0452** | **0.0227** |
| **50** | **<0.0001** | 0.146 | 0.2933 |
| **100** | **<0.0001** | 0.052 | 0.4574 |
| **200** | **<0.0001** | **0.0015** | **0.0004** |
| **400** | **<0.0001** | 0.1515 | 0.4784 |

**Supplementary Table S2.** A comparative summary of the effects of Indium (In), Lanthanum (La), and Cerium (Ce) on key markers. Arrows indicate a significant increase (↑/↑↑/↑↑↑) or decrease (↓/↓↓/↓↓↓) relative to control, corresponding to $p < 0.05$, $p < 0.01$, and $p < 0.001$, respectively, while ↔ indicates no significant change. SOD (Superoxide Dismutase); CAT (Catalase); MDA (Malondialdehyde); IL-6 (Interleukin-6); IL-1β (Interleukin-1β)

| **Cell Line** | **Parameter** | **Indium (In)** | **Lanthanum (La)** | **Cerium (Ce)** |
|---|---|---|---|---|
| **Normal Fibroblasts (BJ)** | **SOD** | ↓↓↓ | ↓ | ↓ |
| | **CAT** | ↔ | ↓↓ | ↑↑↑ |
| | **MDA** | ↑↑ | ↓ | ↓ |
| | **IL-6** | ↑↑ | ↔ | ↓↓↓ |
| | **IL-1β** | ↔ | ↔ | ↔ |
| **Hepatocarcinoma (HepG2)** | **SOD** | ↑↑↑ | ↑↑ | ↑↑↑ |

| | | | | |
|---|---|---|---|---|
| | **CAT** | ↔ | ↔ | ↔ |
| | **MDA** | ↓↓ | ↑↑↑ | ↓↓↓ |
| | **IL-6** | ↓↓↓ | ↑↑↑ | ↓↓↓ |
| | **IL-1β** | ↑↑ | ↔ | ↓ |
